# Supplementary material for: Are carbon emissions trading and green financial instruments synergistic? -Comprehensive quantitative research based on content analysis
Source: PLoS One. 2024 Mar 7;19(3):e0298601. doi: 10.1371/journal.pone.0298601 (PMC10919601; doi:10.1371/journal.pone.0298601)
Supplement: S1 Data — (ZIP) [file pone.0298601.s001.zip › Data/地级市Z_F工作报告.zip!/║╙▒▒/╨╧╠¿╩╨/└┤╫╘╣1⁄2╓┌║┼í╛┬φ┐╦╩2╛▌═°í┐_2016.docx]

各位代表：

　　现在，我代表市政府作《政府工作报告》，请各位代表连同《邢台市国民经济和社会发展第十三个五年规划纲要（草案）》一并审议，并请市政协委员和列席会议的同志提出意见。

　　一、“十二五”时期经济社会发展回顾

　　 “十二五”时期，面对复杂严峻形势和经济下行的巨大压力，在中共邢台市委的坚强领导下，在市人大的法律监督、工作监督和市政协的民主监督下，我们紧紧依靠全市人民，积极适应经济发展新常态，主动作为，克难攻坚，经济社会发展取得新成就。

　　综合实力不断壮大，发展基础进一步夯实。到“十二五”末，全市生产总值达到1764.7亿元、年均增长8.1%，人均生产总值24256元、年均增加1413元；全部财政收入176.5亿元、年均增长5.8%，公共财政预算收入102.7亿元，突破百亿大关，年均增长12.5%；规模以上工业增加值576.7亿元、年均增长9.2%；固定资产投资1825.9亿元、年均增长20.6%；社会消费品零售总额871.8亿元、年均增长13.6%。项目建设持续加力。累计实施市级以上重点项目1072个，完成投资2130亿元，比“十一五”时期增加845亿元，晶澳太阳能、好孩子北方基地等210个重大产业项目竣工投产。累计新增建设用地指标12.5万亩。园区建设实现突破。邢东新区获省政府批准，增添了战略发展平台；省级开发区达到22家，实现县县全覆盖；主营业务收入超百亿元园区达到16个。基础设施建设全面加强。邢台机场完成立项，石武高铁、邯（邢）黄铁路通车，“邢台号”高铁始发运营，邢和铁路动工，京港澳高速改扩建、邢衡高速、邢汾高速竣工通车，全市高速公路通车总里程达到567公里，实现县县通高速；南水北调邢台段建成通水，17个县（市）水厂基本建成，西线引黄工程竣工；邢台热电联产项目开工建设。

　　结构调整扎实推进，产业层次进一步提升。三次产业比重由15.7∶55.6∶28.7调整为15.6∶45.0∶39.4。农业持续增产增效。粮食连年丰收，2015年总产达到451.1万吨，比2010年增加26.9万吨；畜牧、蔬菜、果品占农业总产值比重达到57%，提高9.3个百分点；农业产业化经营率达到67.2%，提高7.2个百分点；新增威县国家现代农业示范区、国家级农业科技园区。工业加快转型升级。坚决化解过剩产能，累计压减炼铁232.5万吨、炼钢152万吨、水泥703.3万吨、玻璃6417.5万重量箱，全市单位GDP能耗累计下降25.8%，高于省定目标6.8个百分点。传统优势产业迈向中高端，累计774个技改项目列入省重点、完成投资1203亿元。装备制造业成为第一支柱产业，增加值占规模以上工业比重达到24.7%。特色产业集群升级加快，清河羊绒、临西轴承等10个集群主营业务收入超百亿元，沙河被认定为全国玻璃期货基准价区。战略性新兴产业特别是汽车及新能源汽车产业加速成长，长征汽车、红星汽车成功实施战略重组，御捷车业发展成为全国小微电动车行业标杆企业，德动碳纤维电动车等项目进展加快，与北汽、中国动力等境内外大企业达成合作，新能源汽车全产业链条正在形成。现代服务业不断发展。全市金融机构存贷款余额分别达到2936.9亿元、1759.2亿元，比2010年分别增加1357.9亿元、945.1亿元，存贷比达到59.9%、提高8.3个百分点；交通银行等4家银行在我市设立分行；新增挂牌上市企业39家。2015年，旅游业总收入139.9亿元，是2010年的3.4倍。天一城、北国商城等商贸综合体建成开业，物流、电商、健康养老等新兴业态发展迅猛，清河、平乡被列为全国电子商务进农村综合示范县。2015年，服务业增加值完成695.5亿元，对经济增长的贡献率超过50%，成为重要拉动力量。

　　城乡统筹日益深入，功能设施进一步完善。全市常住人口城镇化率达到47.73%，提高6.8个百分点。中心城市品质提升。“一城五星”总体规划编制完成并开始实施，建成了连接“五星”的绿色长廊和快速路。龙岗、高铁片区启动开发，七里河片区建成国家级水利风景区。水、路、电、气、讯等功能日益完善，市区集中供热率达到90.1%、提高8.7个百分点。市图书馆基本建成并对外开放，博物馆开工建设，智慧城市建设加快，成功创建国家园林城市。县城镇建设步伐加快。16个县（市）编制完成新一轮城乡总体规划，威县被列为国家新型城镇化综合试点，南和建成国家园林县城，新增省级园林县城13个、卫生城6个，邢家湾等4个镇被列为全省经济发达镇行政管理体制改革试点，一批公园、特色街区等工程建成投用，新增垃圾处理场16座。美丽乡村建设取得成效。建设省级重点村478个，公路、广播电视实现村村通，农村人居环境不断改善。

　　改革创新持续深化，发展活力进一步释放。重点改革深入推进。大力实施简政放权，压减市本级职权2557项、下放342项，推行“先照后证”、注册资本认缴登记制改革，威县作为全省综合改革试点县，创新成立审批局、市场监管局等做法全面推广；深入开展大众创业、万众创新，各类市场主体达到33.2万户，比2010年翻了近一番；市、县政府机构改革基本完成，财政预算绩效管理、农村土地确权颁证等改革不断深化。开放水平不断提高。深入实施“三个一百”活动，组织开展系列专题招商活动，累计利用外资19.3亿美元，卡博特炭黑、中航长征、超威电池等一批投资超10亿元项目落户我市。抢抓京津冀协同发展机遇，实施合作项目156个，总投资1803.6亿元，威县与顺义、沙河与房山、市开发区与通州合作共建园区，市政府与北控、金隅等大型企业签署战略合作协议。邢台海关开关运行，外贸出口总额累计63.3亿美元，是“十一五”时期的1.6倍。科技支撑作用日渐增强。推进质量提升三年行动、百千万人才引进工程，全市研发经费投入占GDP比重达到0.78%、比2010年提高0.17个百分点，每万人拥有发明专利0.586项、翻了一番多，高新技术企业达到102家，建成国家级企业技术中心3家、国家级重点实验室1家、院士工作站14家，引进海外人才100名，获得中国驰名商标26件。

　　污染防治力度空前，生态建设进一步加强。向大气污染宣战。累计投入140多亿元，全面开展污染源排查，深入实施节能、减煤、控车、抑尘等措施，深度治理企业1816家，关停企业1289家，蓝鸟公司、变压器公司等10余家企业搬离市区，全市拆除燃煤锅炉1698台、关停小煤矿70家，环市区小水泥、小石灰、小石子等企业全部取缔，“气化邢台”、型煤配送、公交和出租车油改气、淘汰黄标车等专项行动取得明显成效，积极探索科技治污、第三方治理等新模式，治理机制更加完善，主要污染物排放量大幅削减。2015年，二级及以上天数140天，比2013年增加102天，PM2.5平均浓度比2013年下降36.9%。有效改善水生态。对35座水库实施除险加固，“四河还清”全面推进，对17条境内河流启动全面治理，滏阳河等4条河流出境断面水质达到省考核要求，新增生活污水处理厂25个，地下水超采综合治理完成投资22.81亿元，压采地下水2.36亿立方米，治理水土流失面积484.1平方公里。大力开展造林绿化。新增造林合格面积78.7万亩，森林覆盖率达到26.7%。开发建设生态经济沟591条、123.8万亩，山区综合开发经验在全省推广。在浅山丘陵区大力推进百里优质核桃产业带建设，成功举办首届中国核桃节。扎根我市多年的林业专家李保国被授予“燕赵楷模”称号，省委作出向李保国同志学习的决定。

　　依法治市步伐加快，民生事业进一步改善。加强政府自身建设。把遵守宪法和法律作为施政根本原则，建立政府常务会学法制度，依法决策、民主决策水平不断提高；完善政府工作规则，清理政府文件3298件，压缩审批时限75%；严格落实中央“八项规定”，坚决纠正“四风”，清理处置违规公务用车491辆、超标办公用房7343平方米，处理不作为、乱作为550余人；畅通民意渠道，实行公民列席政府常务会制度，自觉接受监督，认真办理人大代表建议、政协提案5305件，按时办结率100%、满意率98%。社会保障体系日趋完善。民生支出累计1104亿元，占财政总支出的78.6%，比“十一五”时期增加697亿元。城乡居民人均可支配收入分别达到21895元和9152元，年均分别增长11%和13.1%。新增城镇就业31.8万人，转移农村劳动力93万人（次）。城乡居民养老保险制度实现全覆盖，职工工伤保险、失业保险、城镇基本医疗保险实现市级统筹，社保、工资、津补贴水平持续提升。国开行棚改贷款到位28.5亿元，累计开工保障性住房和棚户区改造住房10.7万套，改造农村危房4.9万户。60.1万贫困人口实现稳定脱贫，240万农村人口饮水安全问题得到解决。社会事业繁荣发展。新增幼儿园691所，新建、改扩建中小学856所，新增国家级示范性中职学校3所，建成覆盖主城区的基础教育城域网。市眼科医院冠名河北省眼科医院，组建市儿童医院，新建迁建市级医院6家，县级公立医院综合改革实现全覆盖，乡镇医疗卫生机构改造任务全部完成。群众文化生活丰富活跃，成功承办省第8届残运会暨第4届特奥会、省第17届大运会等重大赛事。社会大局保持稳定。安全生产形势持续好转，食品药品安全工作走在全省前列，“平安邢台”建设深入推进，荣获全国“六五”普法中期先进城市称号，群众安全感不断提升。援疆援藏、双拥共建、统计审计、民族宗教、外事侨务、防震减灾、人防气象、老龄妇幼、残疾人、档案、地方志等事业取得新成绩。

　　刚刚过去的2015年，我们坚持解放思想、抢抓机遇、奋发作为、协同发展，扎实开展“三严三实”专题教育和解放思想大讨论，严守发展、生态、民生三条底线，着力优增量提质量、建平台聚要素、治污染促转型、攻难点保稳定，经济社会发展呈现稳中有进、稳中有新、稳中向好的态势。尽管压产能、治污染影响生产总值增速约2.2个百分点，但全市经济运行换档不掉档、调速不失速，生产总值比上年增长6%，公共财政预算收入增长7.3%，固定资产投资增长10.9%，规模以上工业增加值增长4.2%，社会消费品零售总额增长9.5%，城乡居民人均可支配收入分别增长9.4%、9.7%。2015年各项工作的基本完成，标志着“十二五”规划顺利收官，为“十三五”和全面建成小康社会奠定了坚实基础。

　　各位代表，“十二五”时期是邢台发展进程中极不平凡的五年，是经济持续平稳发展、人民生活不断改善的五年，成就来之不易！这是省委、省政府和中共邢台市委正确领导的结果，是市人大、市政协和社会各界监督支持的结果，是全市人民共同奋斗的结果。在此，我代表市政府，向全市人民，向人大代表、政协委员、各民主党派、工商联、无党派人士和人民团体，向驻邢部队、武警官兵、政法干警和驻邢各单位、各企业，向关心支持邢台发展的海内外朋友，致以崇高的敬意和衷心的感谢！

　　各位代表,在总结成绩的同时，我们也清醒地认识到存在的问题：一是经济下行压力仍然较大，部分“十二五”经济指标没有达到预期，不少企业生产经营困难，政府债务负担较重、财政收支矛盾突出；二是产业结构不优，创新能力不强，大项目、好项目少，转型升级任务紧迫而繁重；三是城镇化水平低，城市建设欠账较多，公共服务、社会事业、民生保障还有不小差距，县域经济发展不均衡，脱贫攻坚压力大；四是大气污染防治形势依然严峻，精准治霾、重化围城还缺乏系统解决方案和根本路径；五是关键领域改革还不到位，发展环境不优，吸引力、竞争力不强，体制机制还需进一步理顺。这些问题，既有宏观形势、基础条件、历史积累的原因，更有精神状态、思维理念、工作作风的因素，个别政府部门和工作人员思想保守、封闭僵化，服务意识不强，执行力不够，不勤不廉、消极腐败现象仍然存在。对此，我们一定高度重视，认真加以解决。

　　二、“十三五”时期的主要目标和任务

　　“十三五”时期，是全面建成小康社会的决胜阶段。全球新一轮科技革命和产业变革蓄势待发，全国经济长期向好的基本面没有改变，将为我市加速崛起、攻坚突破、释放各种比较优势带来广阔空间；京津冀协同发展、“一带一路”、“中国制造2025”、“互联网+”等重大战略深入实施，将为我市建设国家新能源产业基地、产业转型升级示范区和冀中南物流枢纽城市带来重大机遇；供给侧结构性改革，“去产能、去库存、去杠杆、降成本、补短板”政策的不断落地，将为我市调存量、上增量，实现动能转换带来难得的政策和市场“窗口期”；邢台文化底蕴深厚，交通枢纽地位凸显，后发潜力和市场空间巨大，特别是民风淳朴、人心思进，将为“十三五”高点起步、跨越赶超带来无穷的内生动力。因此，我们必须以等不起的紧迫感、慢不得的危机感、争一流的使命感，主动有为、奋发作为，全面开启“十三五”的新征程。

　　“十三五”时期我市经济社会发展的指导思想是：全面贯彻党的十八大，十八届三中四中五中全会和省委八届十二次全会、市委八届八次全会精神，以邓小平理论、“三个代表”重要思想、科学发展观为指导，深入贯彻习近平总书记系列重要讲话精神，按照“五位一体”总体布局和“四个全面”战略布局，坚持创新、协调、绿色、开放、共享的发展理念，适应把握引领新常态，高举发展、团结、奋斗的旗帜，坚守发展、生态和民生三条底线，突出协同发展、转型升级、创新突破、跨越赶超的主基调，坚持稳中求进，全面深化改革开放，以提高发展质量和效益为中心，着力加强结构性改革，大力推进新型工业化、信息化、城镇化和农业现代化，坚决打赢脱贫攻坚战，确保如期全面建成小康社会，加快建设经济强市、美丽邢台，为谱写中华民族伟大复兴中国梦的邢台篇章奠定更加坚实的基础。

　　“十三五”时期我市经济社会发展主要指标设定为28项，其中约束性指标14.5项、预期性指标13.5项。总体目标是“一个全面建成、两个翻番、三个高于、四个明显提升”，即：到2020年如期全面建成小康社会；生产总值比2010年翻一番以上，城乡居民人均可支配收入比2010年翻一番以上；经济保持中高速增长、增长速度高于全省平均水平、生产总值年均增长7.5%左右，发展迈入中高端、质量效益提升幅度高于周边基础相近地区、公共财政预算收入年均增长8.5%左右，环境治理大见成效、空气质量改善程度大大高于以往、PM2.5平均浓度比2013年下降50%以上、力争退出全国空气质量后10名；城市经济和县域经济实力、产业发展层次和水平、城镇化发展质量和水平、基本公共服务能力和水平明显提升。重点抓好六个方面：

　　（一）坚持创新发展，增强经济发展新动力。创新是邢台发展的关键。以科技创新提升竞争力。开展产业、技术、产品创新行动，设立一批成果转化基金，建设一批科技园区、技术交易市场，建立一批重点实验室、工程技术研究中心，形成一批掌握核心技术的创新型产业集群，到2020年研发投入占GDP比重达到2%以上，每万人发明专利拥有量1.5件以上，力争高新技术企业达到200家以上，科技型中小企业达到4000家以上。以大众创业万众创新培育新优势。引进和培育并重，尽快形成有邢台产业特色的领军人才和高技能专业团队，设立科技创新贡献奖，重奖有突出贡献的人才。营造统一规范的创新创业环境，鼓励发展众创、众包、众扶、众筹等新模式，建成省级孵化器10家，争创国家级孵化器。以供给创新扩大有效需求。大力推进供给侧结构性改革，扩大新产品和服务供给，以消费升级带动产业转型，以供给创新释放消费潜力。优化投资结构，对接国家“7+4”重大工程包，增加有效投资，项目规模、质量、效益大幅提升。科学配置用地规模、建设用地指标、金融、财政政策等资源，支持比较优势明显、成长性强、带动作用突出的领域，形成又好又快发展的正拉动。以全面深化改革活跃全局。积极承担国家、省改革试点任务，推广威县改革经验，深化行政审批、财政金融、国有企业、农业农村等改革，释放民营经济和县域经济活力，推动市场主体、民营经济实现倍增。

　　（二）坚持转型发展，构建现代产业新体系。通过主动转型，尽快形成先进制造业和现代服务业共同主导、传统产业与新兴产业双轮驱动的发展格局。培育壮大新兴产业。坚持高端起步、质量优先，推进先进装备制造、新能源新材料、汽车及新能源汽车、节能环保等产业加快发展，支持中钢邢机、晶龙、御捷、中航长征、多氟多红星等企业做大做强，加快邢台县龙冈汽车及高端装备、清河汽摩配件等园区建设，到2020年，先进装备制造业作为第一支柱产业的地位更加突出，战略性新兴产业增加值占规模以上工业的比重达到20%以上，努力建设国家新能源示范城市、全省重要的装备制造业基地、新能源汽车生产基地和节能环保产业基地。调整优化传统产业。有序化解过剩产能，钢铁、煤化工、玻璃、水泥等产业加快绿色化、差异化、高端化改造，食品、生物医药、纺织服装等产业市场竞争力明显增强，实施规上工业、县域经济倍增工程，到2020年，全市规模以上工业企业达到2000家以上，力争建成1个以上千亿元产业园区、4个以上500亿元产业园区，新增5个主营业务收入超百亿元县域特色产业集群，一半以上的县（市、区）生产总值超百亿元。加速发展现代服务业。实施智慧物流、金融服务、电子商务、旅游休闲、健康养老、服务外包等重点行业发展行动计划，抓好市开发区综合物流园区、清河电子商务创业园等重点园区，挂牌上市企业达到120家以上。加强旅游资源整合和基础设施建设，打造大峡谷、云梦山、岐山湖等旅游产业集群。积极发展大健康产业，重点建设沿太行山健康旅游养生产业带、市区健康养老中心等，建成8个大健康产业示范园区，基本形成覆盖全生命周期、特色鲜明、布局合理的产业体系。到2020年，力争服务业增加值占GDP比重达到45%。做强做优现代农业。以高端、高效、生态为方向，构建“一环、两带、三板块、多园区”布局。稳定粮食生产，壮大畜牧、蔬菜、果品三大主导产业，推进一二三产融合发展，加快临西光明产业园等示范项目和61个山区生态经济示范区建设，支持今麦郎、金沙河、富岗、绿岭等龙头企业做大做强，培树一批知名品牌，实现由传统农业大市向现代农业强市的跨越。

　　（三）坚持协调发展，创造区域发展新优势。加快实施以人为核心的新型城镇化，常住人口城镇化率和户籍人口城镇化率达到全省平均水平。优化空间布局。积极推进区划调整，做大做强中心城市。依托青银、京广-京港澳、邯黄-邢临等主干道路建设产业发展带和城镇聚集轴，以京广沿线转型发展示范区、黑龙港流域特色经济发展区、太行山生态经济示范区为重点功能区，以宁晋、威县、清河等12个县城和一批特色小城镇为节点，构建“一城、五星、三轴、三区、多节点”发展框架和网络型空间格局。建设现代化中心城市。提升“一城五星”统筹水平，推进交通、市政、水系、绿化、公共服务一体化发展。中心城区，抓好邢东新区和龙岗、七里河等重点片区开发建设；落实“多规合一”，运用新理念、新技术、新材料，建设产城融合示范区；开展全国文明城市、卫生城市创建活动，让城市更加宜居、更富活力。到2020年，中心城区常住人口达到130万以上。抓好县城攻坚行动。持续实施扩容提质，加快农业人口转移步伐。到2020年，沙河、宁晋、清河、威县城区常住人口达到25万以上，南宫、巨鹿、隆尧达到15万以上，其他建成各具特色的县城，积极稳妥推进撤县设市工作，持续抓好三镇融合，建设一批特色小城镇。扎实推进美丽乡村建设。按照环境美、产业美、精神美、生态美的要求，坚持规划引领、因地制宜，整合资源、加大投入，实施改居、改厕、污水治理、村庄绿化等十二个专项行动，到2020年基本实现美丽乡村全覆盖。强化基础设施保障。邢台机场和威县、柏乡通用机场建成投用。完成邢和铁路建设，谋划实施邢济、石邢邯、邢衡等城际铁路和中心城市轨道交通项目。太行山高速建成通车，积极争取宁常高速项目。发挥南水北调工程作用，谋划实施八里庙水库、青山水库、宁晋泊大陆泽蓄滞洪区项目，加快引黄入冀补淀、地下水超采综合治理等工程建设。优化骨干电网结构，完成邢台特高压变电站及过境线网建设。构建新一代信息基础设施，智慧城市框架基本形成，实现光纤入户进村。

　　（四）坚持绿色发展，建设生态宜居新家园。牢固树立绿水青山就是金山银山的理念。一刻不放松地抓好大气污染防治。坚持精准治霾、依法管控，实行重点行业特别排放限值，推行烟（粉）尘、挥发性有机物总量控制，加强钢铁、焦化、玻璃、水泥、电力等重点行业治理，开展“车油路”及扬尘综合治理。加快“气化邢台”建设，加强煤炭清洁高效利用，市县建成区基本实现集中供热。加强大气污染联防联控，完善区域环境监测预警和信息共享机制。强化生态修复与建设。加强污水源头治理，加快滏阳河、七里河、白马河等重点河流生态修复。到2020年，地表水劣Ⅴ类水质断面减少15%以上，力争建成水生态文明城市。大力实施绿色邢台建设攻坚，全市森林覆盖率达到30%，努力争创国家森林城市。促进资源节约循环利用。实行能源和水资源消耗、建设用地总量和强度“双控制”，推进产业生态化、生态产业化，废弃资源综合利用业增加值翻一番，力争50%以上省级园区实现循环化改造。加强生态文明制度建设。健全生态保护补偿和环境信息公开机制，强化环保行政执法和刑事司法联动，实行生态环境损害责任终身追究制度，努力让邢台青山常在、绿水长流、蓝天永驻！

　　（五）坚持开放发展，抢抓跨越发展新机遇。用好京津冀协同发展和“一带一路”等重大机遇，提升开放深度和广度。打造邢东新区开放发展新平台。按照“产城融合、改革为先、生态发展”的思路，大力发展先进装备制造、新能源及新能源汽车、节能环保等新兴产业和现代物流、电子商务、金融、会展、医养、教育等城市服务业，着力打造转型升级及产城融合示范区、先进装备制造业基地、新能源产业基地和新兴业态孵化基地。到2020年，邢东新区生产总值、全部财政收入分别达到380亿元、50亿元以上，年均分别增长12%以上、15%左右。积极精准对接京津。加快编制协同发展“三区一基地”专项规划，以省市级开发区为重点，高标准规划建设集中承载地和特色微中心，与京津共建产业园区，形成新的技术链和产业链，积极承接科技、教育、卫生等功能转移，借力发展高效生态农业和现代服务业。构建开放型经济大格局。创新招商方式，完善产业链、会展、姓氏宗亲、商会组织等招商渠道和平台，重点围绕“4+2”主导产业，引进一批龙头项目和配套项目。加强外贸出口基地建设，支持外贸出口百强企业发展，积极创新跨境电商等外贸方式。拓展与中亚、东南亚、中东地区合作，鼓励钢铁、玻璃等优势过剩产能走出去，实现由产品输出向产业输出的转变。

　　（六）坚持共享发展，迈进安居乐业新生活。始终把人民对美好生活的向往作为奋斗目标。打好脱贫攻坚战。全面落实“六个精准”要求，大力实施“五个一批”行动计划，把扶贫开发与产业发展、美丽乡村建设、生态旅游、土地整理结合起来，积极实施产业就业、易地搬迁、政策兜底、医疗救助等八项脱贫行动。到2018年底，10个扶贫重点县全部摘帽；到2020年，现行标准下农村贫困人口实现脱贫，绝不落下一个贫困户、一个贫困群众。全面保障和改善民生。多渠道增加居民收入，着力提高中低收入群体收入水平。进一步完善覆盖城乡的公共就业服务体系，建立统一的城乡居民基本医疗保险制度，完善城镇职工和城乡居民基本养老保险制度，推行全民参保计划。健全住房保障和供应体系，发展社会救助、社会福利和慈善事业。全面推进文教、卫生等社会事业。优化教育资源布局，提高教育教学质量，学前教育毛入学率达到90%以上，标准化中小学比例达到80%，省级示范性高中达到28所，高中段教育基本普及，国家级重点中职学校达到18所，邢台学院转型为综合性应用大学，邢台医专升为本科院校。建立覆盖城乡的基本医疗卫生制度，市人民医院新院区投入使用，眼科医院创建三级甲等专科医院。深入开展“善行河北?德润邢襄”道德主题实践活动，积极发掘邢襄文化、郭守敬文化等资源，打造邢白瓷等文化产业集群，提升文化软实力。广泛开展全民健身，组织国家级高水平特色赛事，做大做强自行车运动品牌，打造绿色太行健身休闲产业带。全面加强社会治理。落实“四个最严”要求，建立健全食品药品长效监管机制。持续加强安全生产管理，推进生产安全标准化建设。健全公共安全体系，有效预防和化解矛盾纠纷，完善突发事件应急机制，维护社会和谐稳定。

　　各位代表，“十三五”规划前景美好，“十三五”发展任重道远。我们坚信，经过全市人民五年的不懈奋斗，到2020年，全市生产总值将达到2500亿元以上，公共财政预算收入达到150亿元以上，城乡居民人均可支配收入分别达到34000元和14000元以上。一个经济更加繁荣、社会更加和谐、生态更加优美、人民更加幸福的新邢台必将呈现在邢襄大地上！

　　三、2016年主要工作

　　2016年是“十三五”开局之年。我们要积极应对经济下行带来的严峻挑战，妥善处理“稳增长、调结构、促改革、治污染、惠民生、防风险”的关系，主动把握供给侧结构性改革的市场、政策窗口机遇，负责担当、闯过关口，高点起步、强势开局。全年主要预期目标是：生产总值增长7.5%左右；公共财政预算收入增长7.5%；固定资产投资增长11%；规模以上工业增加值增长6%左右；社会消费品零售总额增长10%以上；实际利用外资增长5%；出口总值增长5%；单位GDP能耗下降3.5%，化学需氧量、二氧化硫、氨氮、氮氧化物排放量分别削减1.5%、2%、1%、2%，PM2.5平均浓度下降8%以上；城乡居民人均可支配收入分别增长8%、8.5%。围绕上述目标，突出做好以下七方面工作：

　　（一）聚焦改革创新，加快转换发展动能。全面贯彻“五大发展理念”，用新理念拓展新思路，用新思维打破体制机制制约，用新路径解决发展难题。更加注重深化改革释放活力，大力实施简政放权，突出抓好“放、管、服”，全面清理规范行政许可中介服务事项，探索行政审批局和政务服务中心共建机制，探索实行“多证合一”；继续抓好威县综合改革和4个省级经济发达镇改革试点，推广成型经验；实施国有企业分类改革、分类监管、分类发展，优化配置国有资源，抓好国有企业改革试点；深化财税金融体制改革，加强政府性债务管理，推进农村信用社综合改革；加快公共事业领域改革，理顺政企、事企关系，扩大政府购买服务范围；扎实做好供销社、不动产统一登记、事业单位及农村等改革；完成公车改革。依法依规妥善处置国企改制、企业退市进园、棚户区改造等遗留问题。更加注重鼓励创新破解要素瓶颈，发挥投融资平台和基金作用，打造邢东新区、农业、林业等一批投融资平台，设立城市发展、产业引导、农业担保等一批基金，完善市场化融资机制；推进顺德集团和各县融资平台市场化转型；新增挂牌上市企业10家以上，推介PPP项目10个以上；加强政银企保对接，拓宽投融资渠道。积极争取用地指标，深入挖掘土地存量，严把用地关口，优化土地配置，提高土地利用率、供地率。加强企业家、高端人才、职工队伍建设，引进高层次人才200名以上。更加注重发挥市场作用培育优质增量，鼓励民间资本进入更多领域，谋划建设创新创业园，培育众创空间5家以上，市场主体数量增长15%以上；引导社会资本投向“不出现重复建设，不增加过剩产能，不产生挤出效应”的领域，集中优势资源支持技术水平、产业关联度高的优质项目，努力实现更高效益、更高质量的增长。更加注重支持实体经济增强竞争优势，开展降本增效专项行动，推进“营改增”、结构性减税、电价、气价改革，降低企业制度性交易、人工、财务、物流等成本，鼓励企业加强管理挖潜增效；大力实施企业帮扶，对有市场、有效益但暂时经营困难的企业，及时提供产品对接、金融支持、要素保障等服务，推动企业复产、达产；完善企业培育机制，新增规模以上工业企业100家以上；引导企业创新制度模式、管理模式、商业模式，深化产学研合作，新增产业技术研究院2家、院士工作站2家、科技型中小企业500家，规模以上高新技术产业增加值增长12%以上。

　　（二）强力启动邢东新区建设，加快提升开发区和项目建设水平。开发区是经济建设和项目招商的主战场，是协同发展和创新创业的大平台。邢东新区，突出规划引领，举全市之力推进，确保“一年起好步，两年出形象，三年成规模”。加快核心区控制性详规编制，同步完善主路网、地下综合管廊、海绵城市和公建项目建设规划与施工设计。总投资160亿元以上，年度完成投资30亿元左右，启动高铁综合交通枢纽、中央生态公园、邢台中学和市第一医院东院区等重点工程，建设泉北大街东延、振兴一路南延、心河路改造3条主干道，实施辛兴铺等棚改项目。加快引进战略投资者，制定新区招商规划和政策，大力引资引智引技，依托京津冀协同发展大平台，全面对接长三角、珠三角等发达地区，在“6?16”省经洽会、厦门投洽会、深圳高交会上，推出邢东新区品牌。省市级开发区，深化体制机制改革，提品质、上规模，建设10家以上特色园中园，新增主营业务收入超百亿元园区2家；加快市开发区申报国家级开发区步伐，积极推进润恒农副产品物流园、华氏达生态总部经济、朗科机器人等产业项目；进一步完善开发区考核办法，建立动态管理机制，严格兑现奖惩，对省级开发区出现全省“后两位”的要严肃追责，对市级开发区出现全市“后两位”的坚决取消资格。

　　开展重大产业项目突破年活动，坚持月通报、季调度、半年观摩和重大项目领导分包等制度，完善项目考评和动态调整机制。抓好29个省重点项目，安排市重点项目260个，年度投资400亿元以上，确保100个项目开工、50个项目投产、50个前期项目取得实质进展。顺应政策导向、市场方向，谋划一批重大基础设施和生态项目，项目储备保持在500项以上。提高项目招商实效，精准对接京津，突出差别竞争，继续开展好“三个一百”活动，加快建设共建园区，加强与金隅、首农、北控、中广核、国科控股、华北油田等大企业集团的合作，抓好34个重大项目建设和104个签约项目落地，新引进京津项目20个以上。完善常态化招商机制，发挥好驻邢各单位、各企业作用，开展以商招商、合作招商，跟踪全国、全球领军企业发展方向，力争引进超亿元项目100个以上。

　　（三）着力推进调整重构，加快产业转型升级步伐。把转型升级作为提质增效、治理污染的结合点和突破点，加减乘除并举，提升产业层次和水平。推动传统产业改造提升。积极去产能，稳妥推进企业兼并重整、债务重组，依法处置“僵尸企业”，实现市场出清，完成好省下达的钢铁、水泥、玻璃产能压减任务。深入推进产业结构优化升级三年行动，抓好百项技改和“两化”深度融合。争取国家化解钢铁过剩产能专项资金，深化与大企业和科研单位的合作，推动钢铁产业高端化，努力建设特钢和特钢产品生产基地。推动化工产业精细化，旭阳、建滔等企业由生产商向生产服务商转变，努力打造绿色能源化工园区。用好“一带一路”政策，推进过剩产能向原材料产地和境外转移。推动新兴产业加速成长。抓好投资13.6亿元的德田塔爱格半导体键合线、投资10.3亿元的巨亚德伺服电机等项目，提升产品档次，延伸产业链条，促进先进装备制造业向智能化迈进。加快投资50亿元的中国动力产业园、投资32亿元的北汽隆威新能源汽车、投资22亿元的御捷新能源汽车、投资10亿元的红星新能源汽车、投资6亿元的多氟多锂电池等项目建设，提高研发能力、配套能力和聚集水平，打造全产业链的新能源汽车基地；抓好中航长征中重卡、福玉特种车等汽车项目。加快投资10亿元的晶澳太阳能100MW光伏电站、投资10亿元的晶龙1GW太阳能电池等项目建设，进一步巩固太阳能电池产能、转换率全球领先地位。推进邢台节能环保产业园、桑德循环经济产业园等项目，建设2家“城市矿产”基地。加快推进“互联网+”行动计划，启动建设智能化工厂20个、农业物联网应用试点1个。推动现代服务业提速提质。新引进1家以上域外金融机构，鼓励金融机构开发微贷等金融产品。推进冀南物流中心等项目建设，加快申报邢台保税物流中心（B型），开通邢台内陆港冀新欧班列。做好跨境电商平台省级首批试点工作，加快发展信息服务、会展经济、服务外包等产业，争创省级服务外包示范城市，打造“北方声谷”。深入挖掘消费潜力，发展住房租赁市场，落实新能源汽车补贴政策，推广新能源标准车2900辆以上。对外贸出口100强企业实施精准支持，新增外贸市场主体100家以上。加快大健康产业发展步伐，出台产业规划和三年行动计划，启动投资50亿元的石药集团生物制药产业园建设，推进内丘中医药养生文化产业基地和巨鹿金银花、枸杞种植基地建设。筹建游客集散中心，加快大峡谷、红石沟、周公山等项目建设，完善重点景区游步道、宾馆、停车场、厕所等配套设施，提升景区通达和接待能力，做好央视旅游形象宣传，旅游业总收入150亿元以上。推动现代农业加快发展。按照“稳粮、压棉、扩蔬、强畜、增果”总体思路，加快调整农业结构，确保粮食总产450万吨以上，畜牧、蔬菜、果品占农业总产值比重达到58%以上。坚持“园区、龙头、品牌、标准”四位一体，加快转变农业发展方式，完善提升4家、新增3家以上省级农业园区，力争每个乡镇建设1家农业园区；培育一批龙头企业，抓好宁晋玉锋玉米深加工、巨鹿乐民食用菌等项目，每县至少引进1个超亿元项目；打造一批知名品牌，高标准建设唐山世界园艺博览会邢台园，扩大富岗苹果、绿岭核桃等品牌影响力；制修订一批市级农业标准，并争取国家、省和行业标准立项；农业产业化经营率提高1.5个百分点。

　　（四）围绕城乡统筹发展，加快新型城镇化进程。突出活力、宜居、特色，提高规划建设管理水平，进一步做优老城、做大新城、做强县城、做美村镇，常住人口城镇化率提高2个百分点以上。提高中心城市宜居度、带动力。落实中心城区品质提升三年行动计划。实施规划编修工程，完成城市总体规划编修及省“多规合一”试点工作，编制市区供热、污水处理等专项规划；做好土地利用总体规划中期调整完善工作。实施功能完善工程，加快总投资7亿元的召马地表水厂、总投资4.5亿元的污水处理二厂等项目建设；抓好邢台热电联产、旭阳安能二期、德龙金昊等供热项目，新建供热主管网57公里，市区新增集中供热面积460万平方米；水上运动公园主体完工，加快博物馆建设，启动中糠大厦、市公安局警务中心等公建项目。实施市区畅通工程，完成滨江路北段、育英街地道桥等改造建设和建设大街地道桥扫尾工程，打通红星街东延、建设大街东延、新华路北延、平安路贯通等断头路，启动实施金泉大街、长青路、学院路、文苑路、太行路北延、团结大街西延、永安路跨七里河大桥等工程，加快社会停车场建设，规范道路两侧停车。实施增水扩绿工程，加快道路游园绿化提升，新增绿化面积21万平方米，推进污水管网及“四河”治理。实施精细管理工程，加快智慧城市建设，推进云计算中心、环保云平台等项目，市区大型商业设施、公交车覆盖免费WiFi，完善城市治理体系，提升物业管理服务水平。大力发展城市经济，积极培育总部经济、楼宇经济等增长点，加快万达广场、天一港、丰基?欢乐世界、顺德新街等商贸综合体建设。同时，加强“一城五星”统筹，完成邢石大道工程，谋划实施新环城公路，推进供热、燃气、供电、环卫等基础设施共享。增强城镇承载力、吸引力。启动县城建设三年攻坚行动，实施总投资700亿元以上的614项重点工程，力争所有县城实现集中供水和污水、垃圾达标处理，县城集中供热和清洁能源供热率达到75%以上。抓好威县国家新型城镇化综合试点，支持有条件的县城争创国家园林县城、森林城市、环保城市，实现省级园林县城全覆盖。加快22个特色小镇发展，继续推进三镇融合。加快户籍制度改革和居住证制度双落地，建立健全“人地钱”三挂钩机制，加快产城教融合发展，推进县域经济增比进位。加快美丽乡村建设。打造省级重点村393个，落实好十二个专项行动，抓好内丘扁鹊大道和沙河白塔、临城西竖等省市重点片区，启动15个中心村建设示范点，力争每个县（市）建成1个美丽小镇。完善重大基础设施。邢台机场、威县、柏乡通用航空机场开工建设；抓好邢和铁路建设，谋划中心城市轨道交通工程；太行山高速全面开工，邢左线、邢临线等完成升级改造，东华路大修、107国道出市口改造和308国道、邢清线改建等开工建设，邢德线、隆昔线等改建项目加快前期；加快推进1000千伏特高压输变电工程，开工建设500千伏邢西变电站；谋划推进八里庙水库、青山水库等大型水利工程，基本完成南水北调地表水厂建设，县县用上长江水。

　　（五）坚决打赢脱贫攻坚战，加快补齐全面小康短板。认真贯彻中央、省扶贫会精神，深入实施精准脱贫攻坚年。创新扶贫开发方式。培育蔬菜、林果、养殖和手工业等脱贫产业，新增林果、设施蔬菜、中药材4万亩以上，家庭手工业专业村50个，每个贫困县至少建设1个农民工返乡创业园。积极开展电商、旅游、生态、康养、光伏电站等新业态扶贫，发展旅游配套服务专业村4个、蔬菜+光伏发电项目专业村10个、电商扶贫专业村20个以上，电商服务站基本实现贫困村全覆盖。提升贫困地区公共服务水平。启动贫困人口易地搬迁，打好脱贫攻坚“当头炮”。加强就业服务，确保贫困家庭劳动力至少掌握一门致富技能。从秋季学期开始，在市内公办普通高中、中职学校、普通高校就读的建档立卡贫困家庭学生，免收学费、住宿费、教科书费，享受国家助学金政策。对贫困人口实施基本医疗保险、大病保险、医疗救助三重医疗保障，提高救助标准。抓好低保线、扶贫线“两线合一”，对完全或部分丧失劳动能力的贫困人口全部纳入农村低保。完善扶贫政策机制。市本级安排扶贫资金比上年增长140%，单列新增建设用地指标1000亩专项用于扶贫项目建设。广泛动员社会力量参与扶贫事业，完善对口帮扶机制，提高定点扶贫成效。增加减贫成效考核权重，加强督查问责，倒排时间、强力推进、务求实效。年内实现14.43万人稳定脱贫，内丘、临西、任县、南和脱贫摘帽。

　　（六）强化科学精准治污，加快改善生态环境。调整能源结构，完善减排措施，狠抓污染治理革命性工程。深入推进大气污染防治攻坚。大力实施“气化邢台”，力争完成“一城五星”15万户煤改气、煤改电工作，全市推广洁净型煤30万吨，净削减燃煤总量25万吨。实施精准治霾，针对重点区域、重点企业，逐一制定治理方案，开展散煤、焦化行业、露天矿山、道路车辆污染整治专项行动，深入治理市区周边小板材厂，关停取缔33家土小企业及重污染企业，完成54家企业挥发性有机物综合治理。强化面源治理，狠抓扬尘治理和秸秆禁烧，推进施工扬尘实时监控，市区道路机械化清扫率达到60%以上，秸秆综合利用率达到95%。推进排污权、碳排放权、节能量交易和生态补偿，推广第三方治污，加快与德国能源署合作步伐，抓好常州循天公司与沙河玻璃行业合作的趋零排放示范工程。建立健全环境监测预警体系，保持环保执法高压态势，完善重污染天气应急响应机制，运用综合措施降低污染危害。切实加强水生态治理。以创建全国水生态文明城市为抓手，深入推进“碧水工程”，开展饮用水源地安全防护专项行动，按质保量完成引黄入冀补淀工程和地下水超采综合治理节点任务，继续实施人工补水，万元GDP耗水量降低5.6%。强力推进水污染整治7大工程，加快污水处理设施建设和提标改造，污水集中处理厂实现工业园区全覆盖，城镇生活污水处理率达到85%；主要河流断面水质达到省考核要求。按照资源化、市场化、法制化、生态化原则，统筹推进河道综合整治。大力开展生态绿化。启动国家森林城市创建和太行山绿化三年攻坚行动，抓好国家储备林基地建设，完成造林绿化34万亩；深化山区综合开发，启动建设山区生态经济示范区24个。实施分类管控，完善土壤污染防治体系。

　　（七）切实保障改善民生，加快发展各项社会事业。按照保基本、兜底线的要求，提高公共服务数量和质量，让人民群众有更多获得感。全面加强社会保障。城镇新增就业6.2万人，城镇登记失业率控制在4.5%以内。整合城乡居民基本医保制度，推进机关事业单位养老保险制度改革，继续提高城乡居民基础养老金、城乡低保、农村五保对象补助水平。推动企业职工工资合理增长，多渠道增加农民收入。开工保障性住房和棚户区改造住房1.87万套以上，基本建成0.8万套，完成农村危房改造3200户。巩固提升农村饮水安全水平。全面发展社会事业。理顺管理体制，改善办学条件，新增标准化中小学150所，北师大邢台附中、北二外邢台外国语学校开工建设，加快临西等5县（市）城域网建设并网步伐，推进职业教育与地方产业深度融合，支持民办教育健康发展。实施全面两孩政策，深化县级公立医院综合改革，加强重大疾病防控和卫生应急救助，加快市人民医院新院区建设，市三院门诊楼主体完工，市五院扩建工程开工建设。加强邢国墓地、天宁寺、唐祖陵、柏人城址等遗产和传统古村落的保护及利用，做好方志资源挖掘和修志编鉴工作，推进紫金山文化产业园等项目建设，推出新版河北梆子《吕玉兰》等文艺精品。建成国家高水平体育后备人才基地，争办中国邢台绿色太行国际公路自行车大赛和全国特奥融合足球比赛。全面维护社会安定和谐。强化安全生产和食品药品安全监管，深入开展油气管网、尾矿库、危险化学品等专项整治，继续实施小煤矿关闭整合，依法有序退出石膏矿，健全火灾防范救援体系，新创建省级食品药品安全县（市、区）13个。强化风险防控，有效化解政府性债务、征地拆迁等领域的风险隐患，继续加大防范和打击非法集资力度，妥善做好化解过剩产能涉及职工转岗和再就业等工作，大力治理拖欠农民工工资问题。加强和创新社会治理，增强突发公共事件应急预警能力，深化“天网工程”建设、“创净土”行动，依法打击各类违法犯罪行为，确保社会大局和谐稳定。同时，推进援疆援藏、双拥共建、统计审计、民族宗教、外事侨务、法制档案、防震减灾、人防气象、老龄妇幼、残疾人等工作取得新进步。

　　四、狠抓工作落实

　　习近平总书记多次强调，一分部署、九分落实。落实是政府工作的生命。各级政府和全体公务员必须强化政治意识、大局意识、核心意识、看齐意识，全面加强自身建设，以更高的效率、更严的标准、更实的措施履职尽责，把各项工作落实到位。

　　（一）激情干事，以更加自觉的精神和境界抓落实。扎实推进“三严三实”专题教育、“两学一做”学习教育，大力弘扬“爱国为民、艰苦创业、求真务实、无私奉献”的玉兰精神，把夙兴夜寐、激情工作作为常态，把实干实政的要求贯穿于思想和行动之中。以心无旁鹜的状态推进工作，围绕“十三五”目标和2016年重点任务，一件事一件事地抓好落实。以百折不挠的毅力攻克难关，对化解过剩产能、推动转型升级、治理大气污染、推进脱贫攻坚等硬任务，拿出有效办法、有力行动，务求取得突破性进展。以事争一流的精神奋发进取，对标先进，找准差距，自加压力，主动对接国家部委、省直部门，积极向上争政策、争资金、争项目、争试点，努力形成比学赶超、争先进位的浓厚氛围。

　　（二）精准发力，以更加过硬的能力和水平抓落实。把解放思想摆在首位，深入学习习近平总书记系列重要讲话精神，全面贯彻“五大发展理念”，认真践行“八破八立”，在联系实际、解决问题上下功夫，在深化改革、推动发展上见实效。加强调查研究，深入基层一线，倾听群众呼声，了解企业所需，针对经济下行、产业转型等带来的困难，制定针对性措施，务求接地气、实打实，不搞表面文章、形式主义。跟踪政策形势，深入研究供给侧结构性改革要求和导向，积极学习借鉴先进地区经验，抢搭重大新政、重大战略、重大商机的快车，在全局中定位、大局中争先，增强工作的前瞻性、实效性。创新方式方法，积极运用改革的思维，打通落实的“最后一公里”，在转换动能、降低成本、补齐短板等重点领域精准突破。

　　（三）优化环境，以更加高效的标准和服务抓落实。把马上就办、办就办好作为基本准则，少开会、开短会，开解决问题的会，凡是定下的事情，上下一心、一鼓作气，真办、快办、办成，不让市场主体和群众的事在自己手中延误，以政府工作的高效释放发展活力。改进服务方式，精简审批前置条件，实施负面清单管理，完善一窗受理、并联审批、网上审批和公共资源交易平台，实行全程代办、跟踪服务，围绕企业和项目所需，加强路、电、水、气、讯等保障，以优质服务降低发展成本。推行政府部门职责信息公开承诺，建立完善企业投诉中心和市长热线，开展作风公开评议，严查涉企违规收费评比检查以及“吃拿卡要”等现象，切实尊重和维护企业、群众合法权益，以良好市场环境增强发展竞争力。

　　（四）从严治政，以更加完善的制度和机制抓落实。推行目标管理，重点工作逐项制订任务书、路线图、时间表，将任务和责任细化到部门、岗位、人头，明确标准要求、时间节点，做到事事有人干、处处有人管、人人有专责。严格督导考核，强化进度公开、结果奖惩，完善情况通报、工作排队、责任倒查等机制，深入开展干部作风整顿年和“一问责八清理”专项行动，狠抓正反典型，严厉整治不能为、不想为、不敢为现象，坚决问责不落实的事、不落实的人。坚持依法行政，做到科学决策、民主决策、依法决策，主动接受人大、政协和社会监督，落实“两个责任”，强化“一岗双责”，把纪律和规矩挺在前面，始终保持惩治腐败的高压态势，努力营造风清气正的政治生态和社会环境。

　　各位代表！新起点孕育新希望，新目标引领新作为。让我们更加紧密地团结在以习近平同志为总书记的党中央周围，在省委、省政府和中共邢台市委的坚强领导下，把思想统一到发展上，把心思集中到发展上，把力量凝聚到发展上，决胜“十三五”，打好翻身仗，为建设经济强市、美丽邢台而努力奋斗！
